# *Drosophila* Syncrip modulates the expression of mRNAs encoding key synaptic proteins required for morphology at the neuromuscular junction

**DOI:** 10.1261/rna.045849.114

**Published:** 2014-10

**Authors:** Suzanne M. McDermott, Lu Yang, James M. Halstead, Russell S. Hamilton, Carine Meignin, Ilan Davis

**Affiliations:** 1 Department of Biochemistry, University of Oxford, Oxford OX1 3QU, United Kingdom

**Keywords:** *Drosophila*, mRNA localization, localized translation, neuromuscular junction

## Abstract

Localized mRNA translation is thought to play a key role in synaptic plasticity, but the identity of the transcripts and the molecular mechanism underlying their function are still poorly understood. Here, we show that Syncrip, a regulator of localized translation in the *Drosophila* oocyte and a component of mammalian neuronal mRNA granules, is also expressed in the *Drosophila* larval neuromuscular junction, where it regulates synaptic growth. We use RNA-immunoprecipitation followed by high-throughput sequencing and qRT-PCR to show that Syncrip associates with a number of mRNAs encoding proteins with key synaptic functions, including *msp-300, syd-1, neurexin-1, futsch, highwire, discs large,* and α*-spectrin*. The protein levels of MSP-300, Discs large, and a number of others are significantly affected in *syncrip* null mutants. Furthermore, *syncrip* mutants show a reduction in MSP-300 protein levels and defects in muscle nuclear distribution characteristic of *msp-300* mutants. Our results highlight a number of potential new players in localized translation during synaptic plasticity in the neuromuscular junction. We propose that Syncrip acts as a modulator of synaptic plasticity by regulating the translation of these key mRNAs encoding synaptic scaffolding proteins and other important components involved in synaptic growth and function.

## INTRODUCTION

Localized translation is a widespread and evolutionarily ancient strategy used to temporally and spatially restrict specific proteins to their site of function and has been extensively studied during early development and in polarized cells in a variety of model systems (Holt and Bullock 2009; Martin and Ephrussi 2009; Meignin and Davis 2010). It is thought to be of particular importance in the regulation of neuronal development and in the plastic changes at neuronal synapses that underlie memory and learning, allowing rapid local changes in gene expression to occur independently of new transcriptional programs (Guzowski et al. 2000; Miller et al. 2002; Ashraf et al. 2006; Lyles et al. 2006; Sanchez-Carbente Mdel and Desgroseillers 2008; Wang et al. 2009; Buxbaum et al. 2014). The *Drosophila* neuromuscular junction (NMJ) is an excellent model system for studying the general molecular principles of the regulation of synaptic development and plasticity. Genetic or activity-based manipulations of synaptic translation at the NMJ has previously been shown to affect the morphological and electrophysiological plasticity of NMJ synapses (Sigrist et al. 2000, 2003; Zhang et al. 2001; Menon et al. 2004, 2009; Pepper et al. 2009). However, neither the mRNA targets nor the molecular mechanism by which such translational regulation occurs are fully understood.

We previously identified CG17838, the fly homolog of the mammalian RNA binding protein SYNCRIP/hnRNPQ, which we named Syncrip (Syp). Mammalian SYNCRIP/hnRNPQ is a component of neuronal RNA transport granules that contain *CamKII*α, *Arc*, and *IP3R1* mRNAs (Bannai et al. 2004; Kanai et al. 2004; Elvira et al. 2006) and is thought to regulate translation via an interaction with the noncoding RNA BC200/BC1, itself a translational repressor (Duning et al. 2008). Moreover, SYNCRIP/hnRNPQ competes with poly(A) binding proteins to inhibit translation in vitro (Svitkin et al. 2013) and regulates dendritic morphology (Chen et al. 2012) via association with, and localization of, mRNAs encoding components of the Cdc-42/N-WASP/Arp2/3 actin nucleation-promoting complex. *Drosophila* Syp has a domain structure similar to its mammalian homolog, containing RRM RNA binding domains and nuclear localization signal(s), as well as encoding a number of protein isoforms. We previously showed that Syp binds specifically to the *gurken (grk)* mRNA localization signal together with a number of factors previously shown to be required for *grk* mRNA localization and translational regulation. Furthermore, *syp* loss-of-function alleles lead to patterning defects indicating that *syp* is required for *grk* and *oskar (osk)* mRNA localization and translational regulation in the *Drosophila* oocyte (McDermott et al. 2012).

Here, we show that Syp is detected in the *Drosophila* third instar larval muscle nuclei and also postsynaptically at the NMJ. Syp is required for proper synaptic morphology at the NMJ, as *syp* loss-of-function mutants show a synaptic overgrowth phenotype, while overexpression of Syp in the muscle can suppress NMJ growth. We show that Syp protein associates with a number of mRNAs encoding proteins with key roles in synaptic growth and function including, *msp-300, syd-1, neurexin-1 (nrx-1), futsch, highwire (hiw), discs large 1 (dlg1),* and α*-spectrin (*α*-spec*). The protein levels of a number of these mRNA targets, including *msp-300* and *dlg1*, are significantly affected in *syp* null mutants. Furthermore, in addition to regulating MSP-300 protein levels, Syp is required for correct MSP-300 protein localization, and *syp* null mutants have defects in myonuclear distribution and morphology that resemble those observed in *msp-300* mutants. We propose that Syp coordinates the protein levels from a number of transcripts with key roles in synaptic growth and is a mediator of synaptic morphology and growth at the *Drosophila* NMJ.

## RESULTS

### Syp is required for synaptic morphology at the *Drosophila* NMJ

**FIGURE 1.**
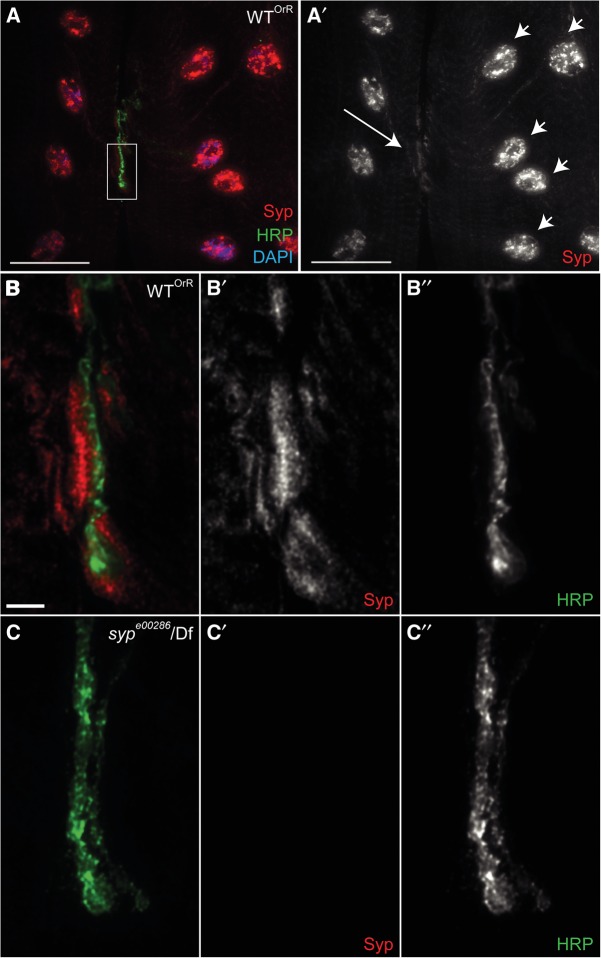
Syp is present throughout the muscle in third instar larvae and is enriched both in muscle nuclei and postsynaptically at NMJs. (*A*) Wild-type Oregon-R (OrR) third instar larval fillet preparations were stained with an anti-Syp antibody (red). anti-HRP antibody was used to label neurons (green) and DAPI used to label muscle nuclei (blue). (*A*′) Syp is present throughout the muscle cytoplasm and is enriched in muscle nuclei (small arrowheads). Syp is also enriched postsynaptically at NMJs (large arrow). (*B–B′′*) The NMJ (white box) highlighted in *A* shown at a higher magnification. (*C–C′′*) The same staining in a *syp* null mutant (*syp*^*e00286*^/Df124) third instar larva. This shows no detectable Syp protein and demonstrates the specificity of the Syp antibody. Images are mean intensity 5-μm projections. Scale bar: (*A*) 40 µm, (*B*,*C*) 5 µm.

The in vivo function of both the mammalian SYNCRIP and *Drosophila* Syp is not well understood in the nervous system. To address this, we first analyzed the Syp protein expression pattern in third instar larvae. We detected Syp in the nervous system of third instar larvae, previously in the brain (McDermott et al. 2012), and postsynaptically at the NMJ in a wild-type third instar larval fillet preparation (Fig. 1A,B). Syp is also present throughout the larval body wall muscles, both in the cytoplasm and particularly enriched in muscle nuclei (Fig. 1A). These signals are not detected in *syp* null mutant larvae, confirming that the protein detected in wild-type larvae is specific to Syp (Fig. 1C).

**FIGURE 2.**
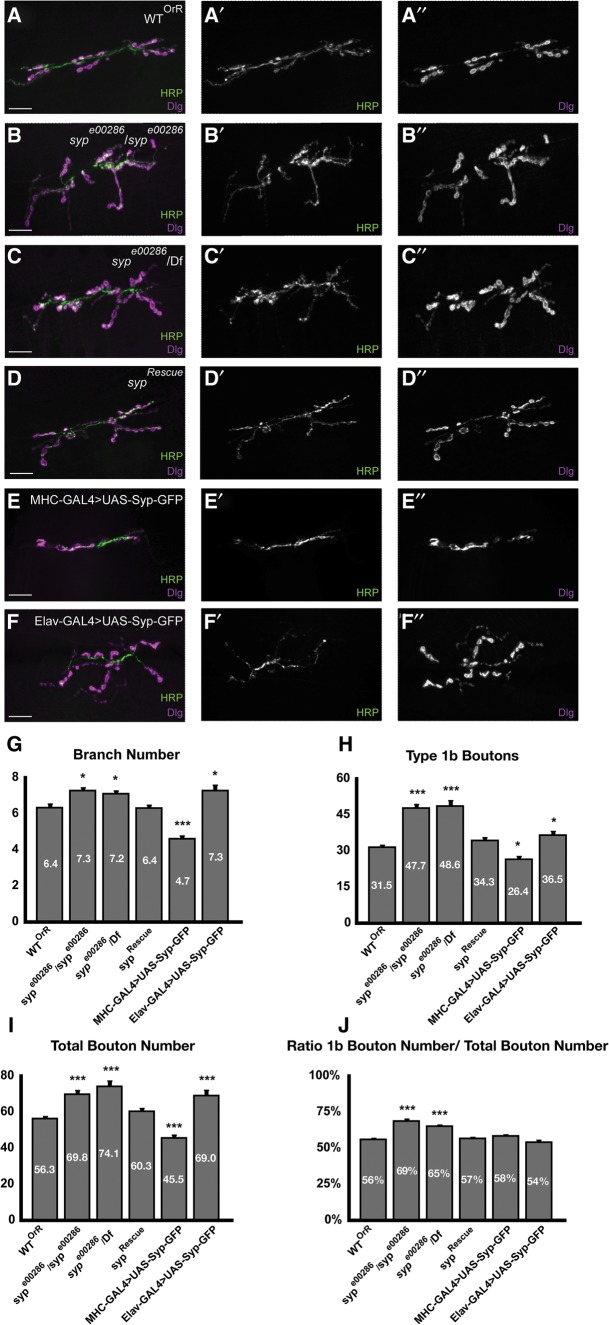
Syp is required for proper growth and morphology of NMJs. (*A*–*F*) Muscle 6/7 NMJs of A3 hemi-segments labeled with anti-DLG (magenta) and anti-HRP (green), showing the degree of branching and number of synaptic boutons. (*A*) Wild-type (OrR); (*B*) *syp*^*e00286*^/*syp*^*e00286*^; (*C*) *syp*^*e00286*^/Df124; (*D*) mutant rescued with a genomic fragment containing all *syp* isoforms except A and H (*syp*^*Rescue*^ is Rescue/Rescue; *syp*^*e00286*^/*syp*^*e00286*^); (*E*) overexpression of Syp in muscle (MHC-Gal4>UAS-Syp-GFP), and (*F*) overexpression of Syp in neurons (Elav-Gal4>UAS-Syp-GFP). Scale bar, 20 µm. (*G*–*J*) Quantification of synaptic structural phenotypes. Error bars indicate the mean ± SEM. Statistical significance was calculated using Student's *t*-test. (*) *P* < 0.05, (**) *P* < 0.01, (***) *P* < 0.001. Numbers of scored NMJs were as follows: OrR, *n* = 31; *syp*^*e00286*^/*syp*^*e00286*^, *n* = 27; *syp*^*e00286*^/Df, *n* = 31; *syp*^*Rescue*^, *n* = 30; Elav-Gal4>UAS-Syp-GFP, *n* = 30; MHCGal4>UAS-Syp-GFP, *n* = 30. In all four graphs, muscle 6/7 NMJs of A3 hemi-segments were analyzed. Muscle sizes for all of the indicated genotypes did not differ significantly from wild type.

Many RNA binding proteins are critical regulators of synapse structure (Sigrist et al. 2000; Zhang et al. 2001; Menon et al. 2004, 2009). To test whether Syp regulates synapse morphology, we characterized the morphology of the NMJs of *syp* mutant larvae. We focused on the highly characterized type 1 NMJs innervating muscles 6 and 7 of the larvae. Wild-type control (OrR) (Fig. 2A) and *syp* null mutant (s*yp*^*e00286*^/*syp*^*e00286*^ or *syp*^*e00286*^/*Df(3R)BSC124*) (Fig. 2B,C; Supplemental Fig. 1A–C) NMJs were stained with anti-HRP to visualize neuronal membrane and with anti-DLG to visualize the postsynaptic scaffold surrounding each type 1b bouton. *syp* mutants show a synaptic overgrowth phenotype, with significantly increased numbers of synaptic branches and boutons, particularly type 1b boutons (Fig. 2G–J), while bouton shape and size were similar in both. While these significant changes in branch (1.2-fold increase) and bouton number (1.5-fold increase in 1b bouton number; 1.3-fold increase in total bouton number) are small, they resemble, and are comparable to, those seen in mutants of other regulators of subsynaptic translation at the NMJ (Menon et al. 2004, 2009). A genomic *syp* rescue construct containing most *syp* isoforms (Fig. 2D) that restores ∼33% wild-type Syp protein expression (data not shown) was able to reverse these phenotypes and restore branch and bouton numbers to those observed in wild-type larvae (Fig. 2G–J).

To test whether excess Syp protein can also lead to a disruption of synaptic morphology, we overexpressed Syp in muscles or neurons using the muscle-specific driver MHC-GAL4 or the pan-neuronal driver Elav-GAL4 (Supplemental Fig. 1A–C). Overexpression in the muscle results in underelaboration of the synapse and generates opposite branching and bouton number phenotypes to those in the *syp* loss-of-function mutant (Fig. 2E,G–J). Moreover, overexpression in the muscle leads to an alteration in the morphology of 1b boutons so that they appear abnormally large and fused (Fig. 2E). In contrast, pan-neuronal overexpression in the CNS using Elav-GAL4 generates a synaptic overgrowth phenotype with significantly increased numbers of synaptic branches and boutons (Fig. 2F–J). We conclude that the levels of Syp protein are important for regulating NMJ synaptic morphology.

**FIGURE 3.**
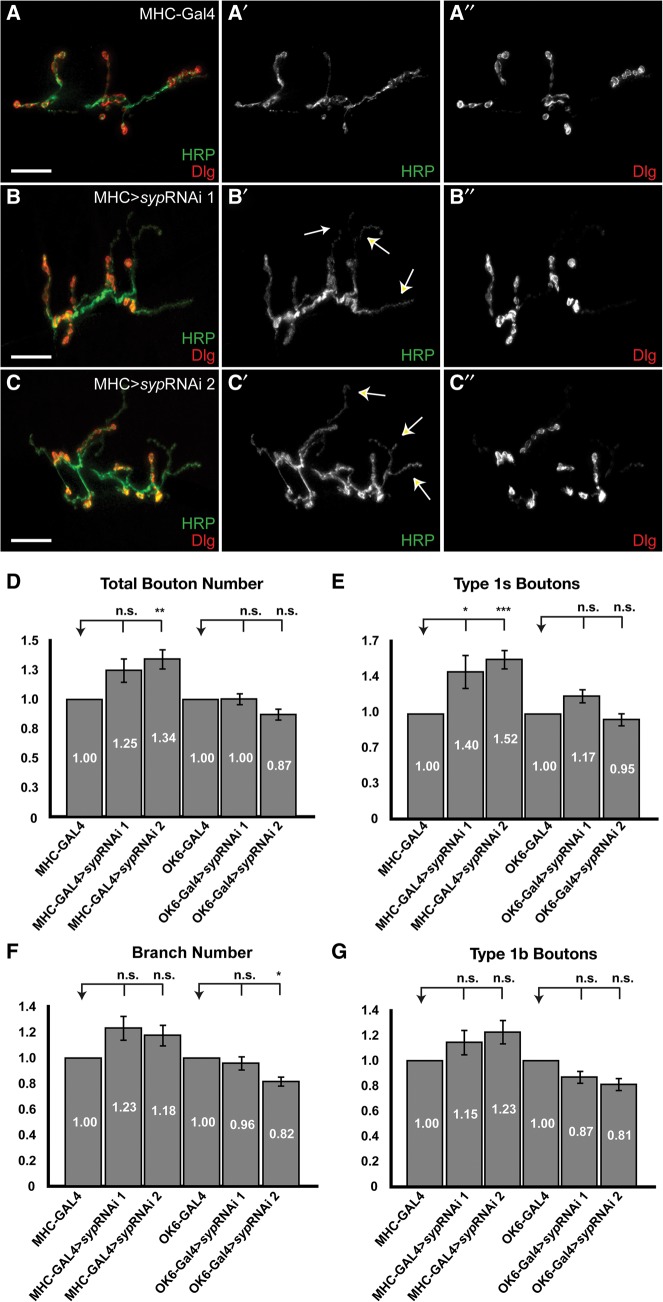
Syp is required in the muscle to suppress NMJ overgrowth. (*A*–*C*) Muscle 6/7 NMJs of A3 hemi-segments labeled with anti-HRP (green) and anti-DLG (red) antibodies in (*A*) MHC-Gal4 third instar larvae, and (*B*,*C*) larvae expressing two independent RNAi insertions in muscle using the MHC-Gal4 driver (MHC-Gal4>*syp*RNAi 1 and 2). Expression of either RNAi construct in muscle leads to overgrowth of the NMJ, with a particular increase in type 1s boutons (arrows). Scale bar, 30 μm. (*D*–*G*) Quantification of synaptic structural phenotypes. Error bars indicate the mean ± SEM. Statistical significance was calculated using Student's *t*-test. (n.s.) *P* > 0.5, (*) *P* < 0.05, (**) *P* < 0.005, (***) *P* < 0.001. Numbers of scored NMJs were as follows: MHC-Gal4, *n* = 27; MHC-Gal4>*syp*RNAi 1, *n* = 24; MHC-Gal4>*syp*RNAi 2, *n* = 24; OK6-Gal4, *n* = 25; OK6-Gal4>*syp*RNAi 1, *n* = 28; OK6-Gal4> *syp*RNAi 2, *n* = 28. In all four graphs, muscle 6/7 NMJs of A3 hemi-segments were analyzed. Muscle sizes for all of the indicated genotypes did not differ significantly from wild type.

In order to clarify the relative requirement for Syp protein post- and presynaptically, we selectively knocked down *syp* expression by driving two *syp* RNAi lines (independent insertions on the second and third chromosomes) with the muscle-specific driver MHC-GAL4 (Supplemental Fig. 2) or the motoneuron-specific driver OK6-GAL4. We found that presynaptic expression of RNAi in motoneurons did not result in a significant decrease in branch number or bouton number (Fig. 3D–G). We conclude that presynaptic *syp* knockdown in motoneurons does not affect synaptic morphology. In contrast, expression of either RNAi insertion in muscle leads to an increase in bouton number (Fig. 3B–G), with a particular increase in type 1s boutons (arrows, Fig. 3B,C). These results, combined with the observed consequences of overexpression of Syp and the fact that Syp is only detectable postsynaptically indicate that Syp is required postsynaptically in muscle to suppress NMJ overgrowth. Interestingly, pan-neuronal Syp overexpression and *syp* RNAi (Supplemental Fig. 3) driven by Elav-GAL4 also result in overgrowth defects in NMJ morphology as assessed by branch and bouton numbers. Therefore, Syp in neuronal cell types other than the motoneurons may also regulate synaptic morphology.

### Syp protein associates specifically with key synaptic transcripts

**FIGURE 4.**
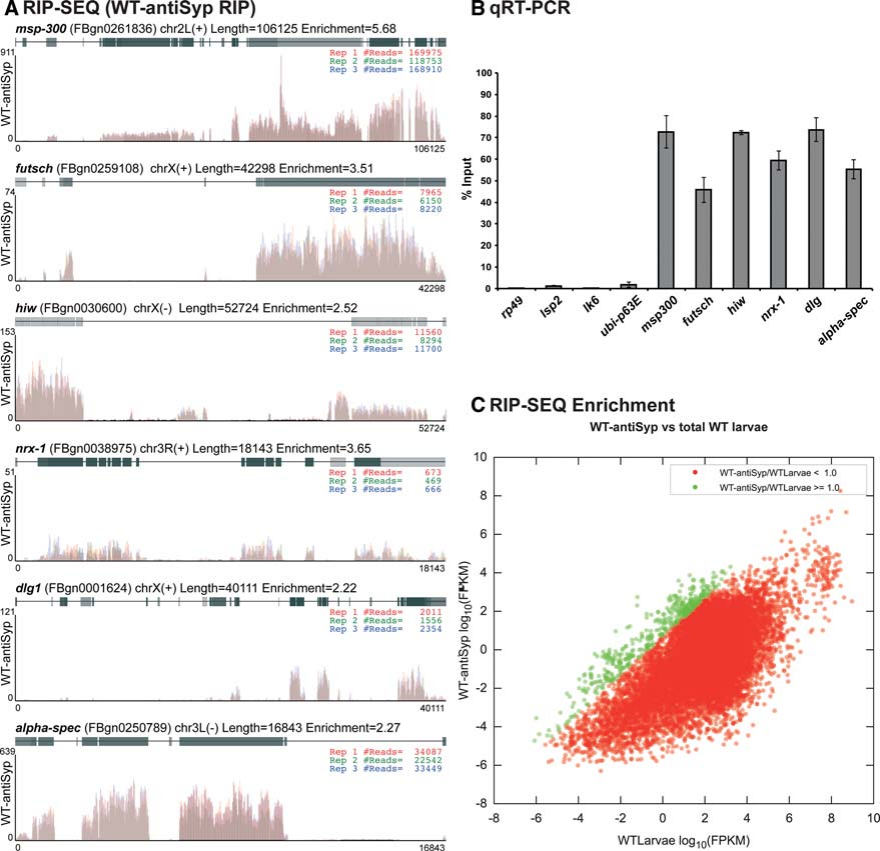
Syp associates with key synaptic transcripts. (*A*) RNA immunoprecipitation (RIP) from larval lysates using an anti-Syp antibody was followed by RNA-Seq to identify RNAs that can associate with Syp. RNA-Seq read coverage plots aligned against gene models for *msp-300*, *futsch*, *hiw*, *nrx-1*, *dlg1*, and α*-spec* for anti-Syp RIP from wild-type larvae (WT-antiSyp). Reads are plotted for each of three RIP replicates in red, green, and blue. The color density of the exons represents the relative occurrence in the known FlyBase transcripts. (*B*) Validation of RNA-Seq results using qRT-PCR following WT-antiSyp RIP. Percent inputs for negative control transcripts that were not enriched (*rp49*, *lsp2*, *lk6*, *ubi-p63E*), and transcripts that were enriched by anti-Syp immunoprecipitation (*msp300*, *futsch*, *hiw*, *nrx-1*, *dlg1*, and α*-spec*) were determined. Data shown represent mean ± SEM from three independent experiments. (*C*) RNA-Seq enrichment plot showing log_2_ FPKM values for WT-antiSyp RIP against total WT larvae RNA-Seq. Points have been colored by enrichment (green, ≥1 and red, <1).

**FIGURE 5.**
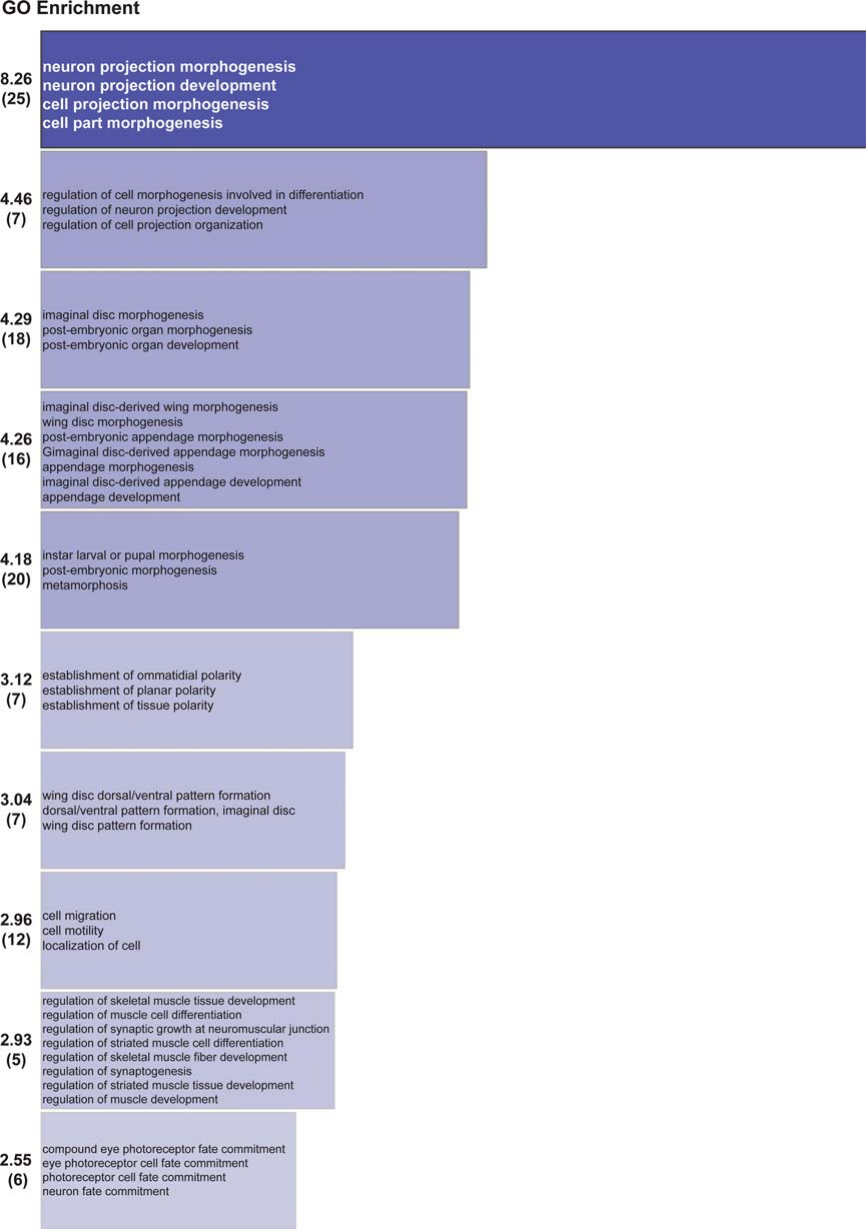
Functional annotation clustering of the Syp RIP-Seq data set. The top ten significant clusters from a DAVID GO Analysis for RNAs with a log_2_ enrichment >1 (see Materials and Methods). Number given is GO enrichment score (number of genes in group).

As Syp is an RNA-binding protein which regulates the localization and translation of mRNAs in the oocyte (McDermott et al. 2012) and is present in RNA transport granules in the dendrites of mammalian neurons (Bannai et al. 2004; Kanai et al. 2004; Elvira et al. 2006; Chen et al. 2012), we sought to determine the downstream mRNA targets of Syp in third instar larvae. Regulation of a number of different RNA targets in different cell types could also account for the complex phenotypes that we observe at the NMJ. We performed RNA-immunoprecipitation (RIP) of native RNP complexes from wild-type (OrR) and *syp* null mutant larval lysate, followed by deep sequencing (RIP-Seq). For a given transcript with a substantial number of fragments per kb of exon model per million mapped reads (FPKM), we calculated the ratio of the number of reads before and after immunoprecipitation. We then focused on those 274 transcripts with a substantial number of reads where there was an enrichment due to immunoprecipitation with anti-Syp antibody from wild-type larval lysate and not in *syp* null mutant lysate (Fig. 4C; Table 1; Supplemental Table 1; Supplemental Fig. 4). Analysis of our RIP-Seq results revealed that Syp associates with a large number of mRNAs encoding proteins with key roles in synaptic growth and function. This was illustrated in the results of a Gene Ontology (GO) functional annotation clustering analysis (Huang et al. 2009a,b) of the 274 RNAs with a log_2_ enrichment >1 (Supplemental Tables 1,2). In this analysis, the top GO annotation cluster corresponds to neuron/cell projection morphogenesis or development (Fig. 5; Supplemental Table 2). *msp-300* (Elhanany-Tamir et al. 2012; Volk 2013; Morel et al. 2014), *syd-1* (Owald et al. 2010, 2012)*, nrx-1* (Li et al. 2007; Zeng et al. 2007; Owald et al. 2012), and *futsch* (Roos et al. 2000; Zhang et al. 2001) were among the top hits, while *hiw* (Wan et al. 2000; McCabe et al. 2004; Wu et al. 2005; Collins et al. 2006)*, dlg1* (Lahey et al. 1994; Budnik et al. 1996; Guan et al. 1996; Tejedor et al. 1997; Thomas et al. 1997), and α*-spec* (Featherstone et al. 2001; Pielage et al. 2005, 2006) were also found to associate with Syp (Fig. 4A; Table 1; Supplemental Table 1; Supplemental Fig. 4). In contrast, abundant transcripts with no known synaptic function in larvae were not associated with Syp. These include *rp49, lsp2*, *lk6*, and *ubi-p63E*. Collectively, these results suggest that the RIP-Seq experiment was successful in identifying specific mRNA that interact with Syp. The RIP-Seq results were further confirmed by qRT-PCR (Fig. 4B).

**TABLE 1.**
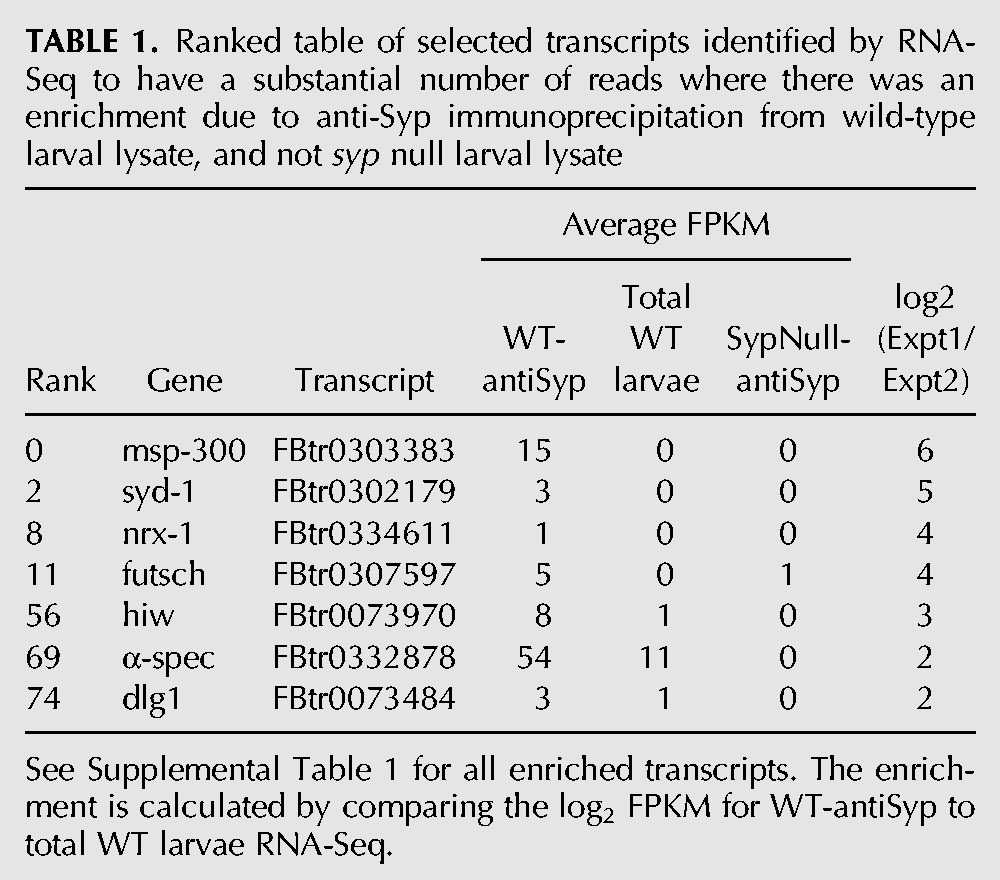
Ranked table of selected transcripts identified by RNA-Seq to have a substantial number of reads where there was an enrichment due to anti-Syp immunoprecipitation from wild-type larval lysate, and not *syp* null larval lysate

### Syp regulates the protein levels of associated transcripts

**FIGURE 6.**
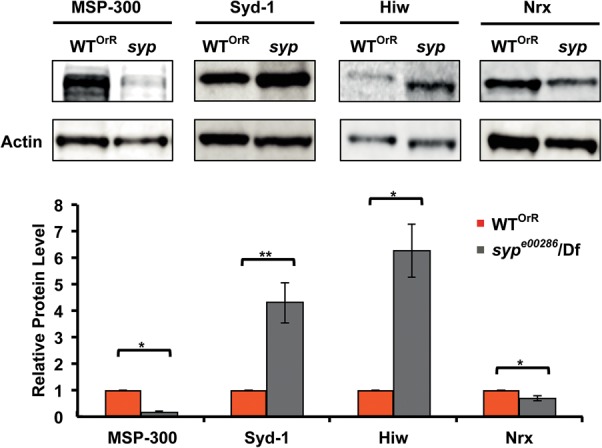
Syp regulates protein levels of associated transcripts. MSP-300, Syd-1, Hiw, and Nrx protein levels in body wall muscle extracts of wild-type Oregon-R (OrR) and *syp* null mutant (*syp*^*e00286*^/Df124) third instar larvae were assessed by Western blot analysis and quantified with the Odyssey imaging system, using actin for normalization.

To further investigate the regulation of Syp target mRNAs identified by RIP-Seq and confirmed by qRT-PCR, we examined the levels of MSP-300, Syd-1, Hiw, Nrx-1, and DLG protein in wild-type (OrR) and *syp* null mutant larval body wall lysates. Our results showed that there are significant changes in the protein levels of these mRNA targets in the body wall of *syp* null mutants; MSP-300 and Nrx-1 are reduced (Fig. 6), while Syd-1, Hiw (Fig. 6), and DLG (both 116-kDa and 97-kDa isoforms) (Supplemental Fig. 5A,B; Mendoza-Topaz et al. 2008) are increased. Conversely, in lysate from larvae overexpressing Syp in muscle, the levels of both DLG isoforms are reduced compared to wild-type controls (Supplemental Fig. 5A,B). Furthermore, at least in the case of DLG, changes in protein levels are observed without changes in *dlg1* mRNA level (Supplemental Fig. 5C). We conclude that Syp is required for the correct levels of proteins that are encoded by the mRNAs with which it associates in RIP, and that loss of Syp can result in differential effects on the levels of target transcripts. In the case of some proteins, Syp functions to keep the protein levels low and in others to keep the protein levels high.

**FIGURE 7.**
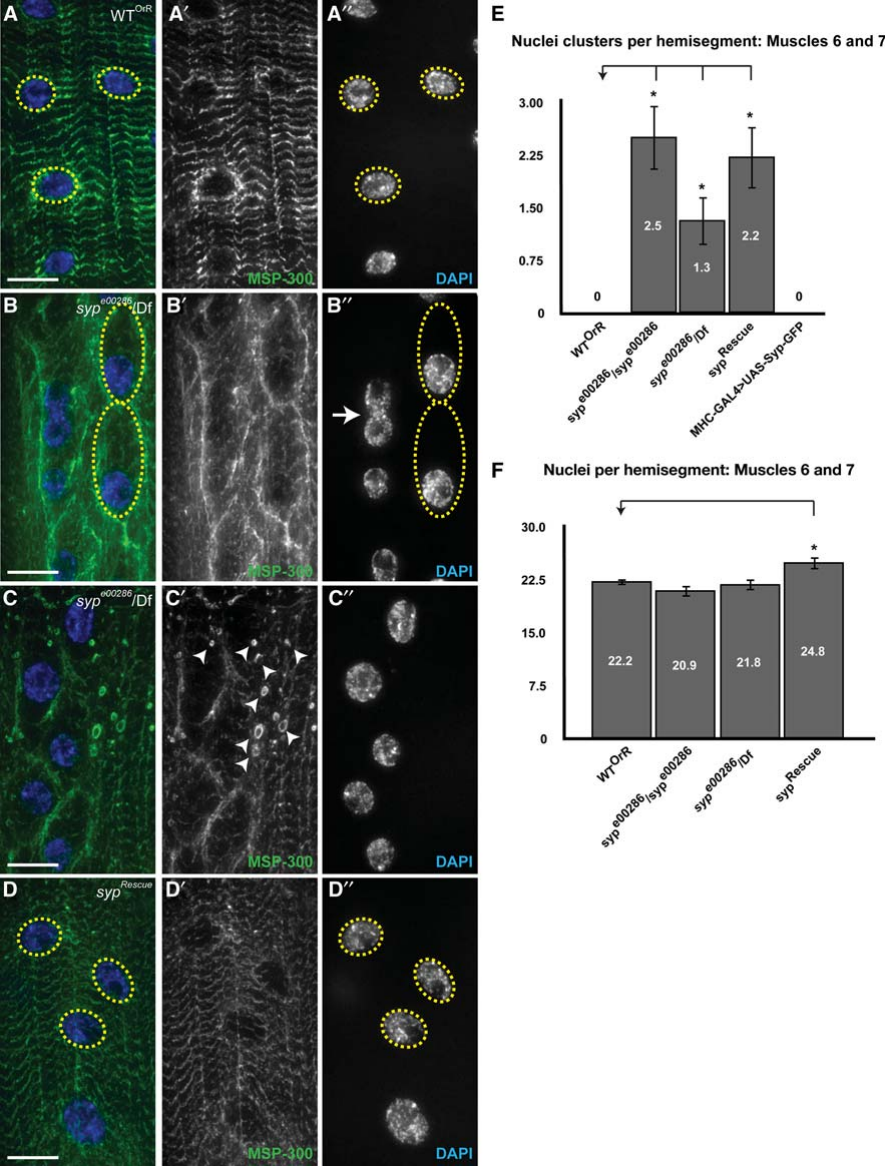
*syp* null mutants have nuclear distribution defects. (*A*) Wild-type Oregon-R (OrR) third instar larval fillet preparations were stained with an anti-MSP-300 antibody (green). DAPI was used to label muscle nuclei (blue). A wild-type distribution of MSP-300 is typified by a striated pattern throughout the myoplasm and a nuclear ring that tightly fits the muscle nuclei circumference (yellow circles). (*B*) The same staining in a *syp* null mutant (*syp*^*e00286*^/Df124) third instar larva. *syp* mutants exhibit a decrease in striated MSP-300 staining and an elongation of the nuclear ring beyond the nuclei circumference. Muscle nuclei are misplaced in *syp* mutants. (*C*) *syp* null mutant (*syp*^*e00286*^/Df124) third instar larvae also exhibit ectopic expression of MSP-300 in discrete circular structures. (*D*) Expression of a genomic fragment containing most all *syp* isoforms (*syp*^*Rescue*^) in the *syp* mutant restores the MSP-300 distribution to wild type, though MSP-300 levels still appear slightly reduced. Scale bar, 40 μm. (*E*,*F*) Quantification of nuclear clustering phenotypes. Error bars indicate the mean ± SEM. Statistical significance was calculated using Student's *t*-test. (*) *P* < 0.05. Nuclear clustering is increased in *syp* mutants relative to wild-type controls, though the number of nuclei per muscle remains unchanged.

*msp-300* was the most highly enriched transcript following anti-Syp immunoprecipitation observed in RIP-Seq (∼5.68-log_2_ enrichment following immunoprecipitation), and MSP-300 protein levels are markedly reduced in *syp* null mutant larval body wall lysates as compared to wild type. To further assess the effect of loss of Syp on MSP-300 function, we stained wild-type (OrR) and *syp* null mutant third instar larval fillet preparations with an anti-MSP-300 antibody. MSP-300 is highly expressed in striated muscles of wild-type third instar larvae (Fig. 7A), and previous studies have indicated that its distribution in the myoplasm reflects an association with the Z-discs (Elhanany-Tamir et al. 2012; Morel et al. 2014). MSP-300 is also detected in a ring-like structure that surrounds each myonucleus (Fig. 7A). Consistent with the reduction in MSP-300 levels that we observed in Figure 6, *syp* mutant larvae exhibit a decrease in myoplasmic Z-disc MSP-300 staining, an elongation of the nuclear ring beyond the circumference of each myonucleus (Fig. 7B), and ectopic expression of MSP-300 in discrete circular structures (Fig. 7C). *syp* mutant larvae also display myonuclear clustering and aberrant nuclear shape and size without change in number (Fig. 7B,E,F). These defects in nuclear distribution and morphology resemble those seen in *msp-300* mutants (Elhanany-Tamir et al. 2012; Morel et al. 2014) and potentially reflect the requirement for Syp in maintaining the correct level and localization of MSP-300 protein. In particular, the MSP-300 nuclear ring staining in *syp* mutant larvae is reminiscent of that observed in an *msp-300* mutant in which exons encoding the C-terminal KASH domain are deleted (Elhanany-Tamir et al. 2012). The *msp-300* gene produces KASH as well as non-KASH isoforms, and in the *msp-300*^▵*KASH*^ mutant, nuclear ring staining was attributed to non-KASH sequences (the antibody recognizes the spectrin repeat domain) that had dissociated from the nuclear envelope. It is possible that the staining and phenotype we observe are due to the regulation of specific *msp-300* isoforms by Syp. A genomic *syp* rescue construct was able to restore MSP-300 distribution to wild type. However, MSP-300 levels still appeared slightly reduced (Fig. 7D), and the nuclear clustering phenotype was not rescued (Fig. 7E). This is possibly due to the caveat that the genomic *syp* rescue fragment does not fully rescue Syp protein expression (∼33% of wild-type levels) (data not shown) and does not contain all *syp* isoforms. A small number of annotated isoforms are not present in the rescue construct, suggesting that missing isoforms are required for full MSP-300 expression and function. This interpretation is consistent with the fact that multiple mammalian Syp isoforms are also required for the correct expression of single target proteins. For example, three mammalian hnRNPQ isoforms can have differential effects on the splicing of *smn* mRNA (Chen et al. 2008).

*msp-300*^▵*KASH*^ mutants display a locomotion defect and a significant decrease in GluRIIA fluorescence density compared to the wild-type control at the NMJ (Morel et al. 2014). To address whether *syp* mutants also have decreased GluRIIA density at the NMJ, we stained wild-type (OrR) and *syp* null mutant third instar larval fillet preparations with an anti-GluRIIA antibody. We detected no difference in distribution or levels of GluRIIA in the *syp* mutant (Supplemental Fig. 6). GluR complexes are also appropriately apposed to active zones in *syp* mutants (Halstead et al. 2014). That we do not detect a reduction in GluRIIA could be due to a number of reasons; first, that Syp regulates MSP-300 function in nuclear positioning and anchoring in the myoplasm but not MSP-300 function in controlling GluRIIA density at the NMJ. Indeed, it has previously been shown that these roles of MSP-300 are distinct and can be disconnected (Morel et al. 2014). It is possible that this is a reflection of distinct functions for multiple MSP-300 isoforms and of the regulation of specific *msp-300* isoforms by Syp as suggested by the pattern of MSP-300 immunostaining in *syp* mutants (Fig. 7). The second possible reason could be that changes in the expression of one or a number of Syp target transcripts in the *syp* mutant, in turn, affect GluRIIA expression. This may mask any differences that we may have seen in GluRIIA levels due to MSP-300 alone. Given that Syp associates with so many key regulators of NMJ function, the NMJ phenotype in *syp* mutants likely represents a complex function of multiple regulators.

## DISCUSSION

*Drosophila* Syp is present throughout the larval body wall muscles, both in myonuclei and in the cytoplasm, and is enriched postsynaptically at the NMJ. Our results demonstrate that Syp is required for the appropriate branching of the motoneurons and the number of synapses they make at the muscle. These observations are potentially explained by our finding that Syp is also required for the correct level of expression of *msp-300, dlg1* and other mRNA targets. Given that we have previously shown that Syp regulates mRNA localization and localized translation in the *Drosophila* oocyte (McDermott et al. 2012), and studies by others have shown that mammalian SYNCRIP/hnRNPQ inhibits translation initiation by competitively binding poly(A) sequences (Svitkin et al. 2013), we interpret these functions of Syp as occurring at the level of translational regulation of the mRNAs to which Syp binds. Our data are also consistent with other work in mammals showing that SYNCRIP/hnRNPQ is a component of neuronal RNA transport granules (Bannai et al. 2004; Kanai et al. 2004; Elvira et al. 2006) that can regulate dendritic morphology via the localized expression of mRNAs encoding components of the Cdc-42/N-WASP/Arp2/3 actin nucleation-promoting complex (Chen et al. 2012).

Translation at the *Drosophila* NMJ is thought to provide a mechanism for the rapid assembly of synaptic components and synaptic growth during larval development, in response to rapid increases in the surface area of body wall muscles or in response to changes in larval locomotion (Sigrist et al. 2000, 2003; Zhang et al. 2001; Pepper et al. 2009). The phenotypes we observed here resemble, and are comparable to, those seen when subsynaptic translation is altered genetically or by increased locomotor activity (Sigrist et al. 2003; Menon et al. 2004, 2009). In *syp* null mutants, NMJ synaptic terminals are overgrown, containing more branches and synaptic boutons. Similarly, bouton numbers are increased by knocking down Syp in the muscle using RNAi. In contrast, overexpression of Syp in the muscle has the opposite phenotype, resulting in an inhibition of synaptic growth and branching. Furthermore, expressing RNAi against *syp* in motoneurons alone does not result in a change in NMJ morphology, indicating that Syp acts postsynaptically in muscle, but not presynaptically at the NMJ to regulate morphology. Interestingly, pan-neuronal *syp* knockdown or overexpression using Elav-GAL4 also results in NMJ growth defects, revealing that some of the defects observed in the *syp* null mutant may be attributed to Syp function in neuronal cell types other than the motoneurons, such as glial cells, which are known to influence NMJ morphology (Berger et al. 2007; Fuentes-Medel et al. 2012). Finally, while Syp is not required in the motoneuron to regulate synapse growth and is not detected in the motoneuron (Halstead et al. 2014), we cannot exclude the possibility that Syp is present at low levels in the presynapse and regulates processes independent of synapse morphology. A further detailed characterization of the cell types and developmental stages in which Syp is expressed and functions is required to better understand the complex phenotypes we observe.

RNA binding proteins have emerged as critical regulators of both neuronal morphology and synaptic transmision (Sigrist et al. 2000; Zhang et al. 2001; Menon et al. 2004, 2009), suggesting that protein production modulates synapse efficacy. Consistent with this, we have shown in a parallel study that Syp is required for proper synaptic transmission and vesicle release and regulates the presynapse through expression of retrograde Bone Morphogenesis Protein (BMP) signals in the postsynapse (Halstead et al. 2014). In this role, Syp may coordinate postsynaptic translation with presynaptic neurotransmitter release. These observations provide a good explanation for how Syp influences the presynapse despite being only detectable in the postsynapse. Here, we have shown that Syp associates with a large number of mRNAs within third instar larvae, many of which encode proteins with key roles in synaptic growth and function. Syp mRNA targets include *msp-300, syd-1, nrx-1, futsch, hiw, dlg1*, and α*-spec.* Syp negatively regulates Syd-1, Hiw, and DLG protein levels in the larval body wall but positively regulates MSP-300 and Nrx-1 protein levels. Dysregulation of these multiple mRNA targets likely accounts for the phenotypes we observe. Postsynaptically expressed targets with key synaptic roles that could explain the synaptic phenotypes we observe in *syp* alleles include MSP-300 (Elhanany-Tamir et al. 2012; Morel et al. 2014), α-Spec (Pielage et al. 2006), and DLG (Lahey et al. 1994; Budnik et al. 1996; Guan et al. 1996; Tejedor et al. 1997; Thomas et al. 1997; Zito et al. 1997; Chen and Featherstone 2005). For example, mutants in *dlg1* and mutants where postsynaptic DLG is destabilized or delocalized have NMJ morphology phenotypes similar to those observed upon overexpression of Syp in the muscle (Koh et al. 1999; Zhang et al. 2007; Bachmann et al. 2010). Presynaptically expressed targets include *syd-1*, *nrx-1,* and *hiw* (Wu et al. 2005; Owald et al. 2010, 2012; Tian et al. 2011; Xiong et al. 2012). However, we have shown that *syp* knockdown in presynaptic motoneurons does not result in any defects in NMJ morphology. The RIP-Seq experiments were carried out using whole larvae and will, therefore, identify Syp targets in a range of different tissues and cells, the regulation of which may or may not contribute to the phenotype we observe in *syp* mutants. It is, therefore, possible that Syp associates with these presynaptic targets in other neuronal cell types such as the DA neurons of the larval peripheral nervous system. It is also possible that Nrx-1 or Hiw are expressed and required postsynaptically in the muscle, but this has not been definitively determined (Wu et al. 2005; Chen et al. 2010). *syp* alleles may provide useful tools to examine where key synaptic genes are expressed and how they are regulated.

The identity of localized mRNAs and the mechanism of localized translation at the NMJ are major outstanding questions in the field. To date, studies have shown that *GluRIIA* mRNA aggregates are distributed throughout the muscle (Currie et al. 1995; Karr et al. 2009; Ganesan et al. 2011). The Syp targets we have identified here, such as *msp-300, hiw, nrx-1*, α*-spec*, and *dlg1*, are now excellent candidates for localized expression at the NMJ. Ultimately, conclusive demonstration of localized translation will involve the visualization of new protein synthesis of targets during activity-dependent synaptic plasticity. Biochemical experiments will also be required to establish the precise mode of binding of Syp to its downstream mRNA targets, the basis for interaction specificity, and the molecular mechanism by which Syp differentially regulates the protein levels of its mRNA targets at the *Drosophila* NMJ. Despite the fact that mammalian SYNCRIP is known to associate with poly(A) tails, our study and other published work have revealed that Syp can associate with specific transcripts (Chen et al. 2012; McDermott et al. 2012). How Syp associates with specific mRNAs is unknown, and future studies are needed to uncover whether the interaction of Syp with specific transcripts is dictated by direct binding of the three Syp RRM RNA binding domains or by binding to other specific mRNA binding proteins. It is also possible that specific mRNA stem–loops, similar to the *gurken* localization signal, are required for Syp to bind to its mRNA targets.

Our study shows that *msp-300* is the most significant mRNA target of Syp. MSP-300 is the *Drosophila* ortholog of human Nesprin proteins. These proteins have been genetically implicated in various human myopathies. For example, Nesprin/Syne-1 or Nesprin/Syne-2 is associated with Emery-Dreifuss muscular dystrophy (EDMD) (Puckelwartz et al. 2009) as well as severe cardiomyopathies (Puckelwartz et al. 2010). Moreover, Syp itself is increasingly linked with factors and targets that can cause human neurodegenerative disorders. Recent work has revealed that SYNCRIP/hnRNPQ and Fragile X mental retardation protein (FMRP) are present in the same mRNP granule (Chen et al. 2012), and loss of expression of FMRP or the ability of FMRP to interact with mRNA and polysomes can cause cases of Fragile X syndrome. Separate studies have also shown that SYNCRIP interacts with wild-type survival of motor neuron (SMN) protein but not the truncated or mutant forms found to cause spinal muscular atrophy (Rossoll et al. 2002), and Syp genetically interacts with *Smn* mutations in vivo (Sen et al. 2013). Understanding Syp function in the regulation of such diverse and complex targets may, therefore, provide new avenues for understanding the molecular basis of complex disease phenotypes and potentially lead to future therapeutic approaches.

## MATERIALS AND METHODS

### Genetics

Stocks were raised on standard cornmeal-agar medium at 25°C. The wild-type (WT) control strain was Oregon R (OrR). The allele *syp*^*e00286*^ is the PBac{RB}CG17838^e00286^ insertion line (Exelixis Collection at Harvard). *syp* mutant third instar larvae were *syp^e00286^/Df(3R)BSC124* (*Df(3R)BSC124*; Bloomington Deletion Project, Bloomington Stock Centre).

The *syp* genomic rescue construct was generated using the clone FlyFos024580 from a genomic fosmid library in the pFlyFos vector (Ejsmont et al. 2009). This clone covers all *syp* isoforms except A and H and was integrated using ΦC31-mediated site-specific recombination into the attP40 site on chromosome II (stock 25709; Bloomington Stock Center) and were generated using the services of Genetic Services Inc.

GAL4 drivers were ElavC155-GAL4 (Bloomington Stock Center), MHC82B-GAL4 (Schuster et al. 1996), and OK6-GAL4 (Aberle et al. 2002; Sanyal 2009). C-terminally GFP-tagged *syp* cDNA was cloned into pUASt for somatic expression in larvae. Several independent transgenic lines were obtained following transformation with this plasmid, and UASt-SypGFP (chromosome III) was used in this study.

*syp* RNAi lines were stocks 33011 (line 1) and 33012 (line 2) (Vienna Drosophila RNAi Center).

### Antibodies

All antibodies used in this study are described in Supplemental Material.

### Immunoprecipitation

Guinea pig anti-Syp, rabbit anti-GFP (Invitrogen), and control pre-immune sera were cross-linked to Protein A Sepharose using standard protocols and the beads equilibrated in IP buffer (50 mM Tris-HCl, pH 8.0, 150 mM NaCl, 0.5% NP-40, 10% glycerol, and Complete EDTA-free protease inhibitor). Twenty microliters of a 50% slurry were added to 500 μg precleared lysate and the mixture incubated overnight at 4°C with mixing. The beads were then rinsed once with cold IP buffer and washed four times for 5 min each at 4°C. After the final wash, the beads were resuspended in 25 μL protein sample buffer (Invitrogen), boiled for 5 min, and the supernatant loaded onto a gel for SDS-PAGE and Western blotting. For RNase treatment, the incubation and wash steps were carried out in the presence of 200 μg/mL RNase A or 0.12 units/μL RNasin Ribonuclease Inhibitor (Promega).

For RNA immunoprecipitation, all steps were carried out in the presence of RNasin, and immunocomplexes were eluted from the beads by the addition of 100 μL extraction buffer (50 mM Tris-HCl, pH 8.0, 10 mM EDTA, and 1.3% SDS) plus 100 units RNasin and incubated for 30 min at 65°C. Following centrifugation, the elution step was repeated and the two supernatants combined. RNA was extracted from input samples and immunoprecipitates using Trizol LS reagent (Invitrogen). Following DNase (Ambion) treatment, immunoprecipitated RNA and 10% total RNA were then used as a template for cDNA synthesis in combination with SuperScript III (Invitrogen) RT and random hexamer primers (Invitrogen). cDNA was then processed for sequencing (RNA-Seq) using the Illumina Genome Analyzer IIx platform (Genomics Services Group, Wellcome Trust Centre for Human Genetics, University of Oxford) or used directly as a template for real-time PCR.

### RNA-Seq data analysis

Alignment of the RNA-Seq reads to the *Drosophila* genome (Release 3) was performed by the sequencing facility using the Stampy alignment tool (version 1.0.22) (Lunter and Goodson 2011). BAM format files were created by the Oxford University Genomics Services Group. The analysis of the RNA-Seq data was performed with a custom-written Perl script (freely available under the GPL license from http://www.darogan.co.uk/RNA-Seq). Calls to SAMtools (version 0.1.18) (http://samtools.sourceforge.net) for reading the BAM sequencing files are made via the Bio-SamTools Perl Module (version 1.33) (http://search.cpan.org/~lds/Bio-SamTools/). Input files for the script are the RNA-Seq BAM files (each experiment in triplicate), chromosome sequences (*D. mel* release 3), and GFF format Flybase annotation file (*D. mel* release 5.49). For each BAM file, the reads are extracted for each annotated gene region. The reads are then assigned to the exons of annotated transcripts for the gene. Fragments per kilobase of exon model per million mapped reads are calculated for paired-end read experiments as defined in Mortazavi et al. (2008). The output from the program is a ranked table of all transcripts above a defined cut-off for RNA enrichment (Table 1; Supplemental Table 1). The enrichment is calculated by comparing WT anti-Syp to WT larvae using log_2_ FPKM values (Zhao et al. 2010). Coverage statistics were calculated using SAMtools and Picard-tools (http://picard.sourceforge.net/version 1.89) and are shown in Supplemental Table 3. The Perl script was also used to generate custom images for selected gene models, showing the WT-anti-Syp IP reads aligned to the gene models. The images are generated as scalable vector graphics (SVG).

The Gene Ontology analysis was performed using DAVID (http://david.abcc.ncifcrf.gov/version 6.7) (Fig. 5; Supplemental Table 2; Huang et al. 2009a,b). A functional annotation analysis was performed on the 274 RNAs with a log_2_ enrichment >1 and compared to a background of the *Drosophila* genome.

### Real-time quantitative PCR

Real-time PCR was carried out using iQ SYBR Green Supermix (Bio-Rad Laboratories) with a Qiagen Rotor-Gene Q according to the manufacturer's instructions. Ten percent of the RT product was used as a template for real-time PCR analysis. Cycle threshold (C[_T_]) values were determined by the second differential maximum method as calculated by the Rotor-Gene software. Calculation of relative mRNA levels was done by using the 2 [-ΔΔC(_T_)] method (Livak and Schmittgen 2001), where the C(_T_) values of the mRNA level were normalized to the C(_T_) values of *rp49* mRNA in the same sample and mRNA levels represented as relative fold change over control. C(_T_) values used were the means of triplicate repeats. Experiments were repeated three times. RNA immunoprecipitation data were also analyzed using the comparative C(_T_) method, factoring in the 10-fold difference in RNA used for input and immunoprecipitation. For each mRNA and each experimental condition, results are expressed as a percentage of the total input RNA. All primers used in this study are listed in Supplemental Table 6.

### Western blotting

Following electrophoresis, proteins were transferred to nitrocellulose (Schleicher and Schuell), or to polyvinylidene fluoride (PVDF) (Bio-Rad) membranes using the XCell II Blot Module (Invitrogen) according to the manufacturer's instructions. Western blotting was performed using standard protocols. Visualization of reactive proteins was performed by enhanced chemiluminescence and quantitative infrared imaging (LI-COR Odyssey, LI-COR Biosciences). Intensity of protein bands was quantified using NIH ImageJ and LI-COR software.

### Immunohistochemistry

For immunostaining, third instar larvae were dissected in HL-3 without calcium (Verstreken et al. 2008) and fixed for 20 min in 4% paraformaldehyde (Thermo Scientific).

### Imaging and de-convolution

A Leica SP5 confocal microscope and a wide-field DeltaVision microscope (Applied Precision, Olympus IX70, and Roper Coolsnap HQ) were used to image fixed material. Live imaging at the NMJ in third instar larvae was performed on a bespoke built upright DeltaVision according to Parton et al. (2010). DeltaVision images were acquired with Olympus 20x/0.75, 40x/0.95, 60x/0.9 or 100x/1.4 objective lenses and then de-convolved (Parton and Davis 2006).

## SUPPLEMENTAL MATERIAL

Supplemental material is available for this article.

## Supplementary Material

Supplemental Material

Companion Paper
