# Supplementary material for: Drosophila Syncrip modulates the expression of mRNAs encoding key synaptic proteins required for morphology at the neuromuscular junction
Source: RNA. 2014 Oct;20(10):1593–606. doi: 10.1261/rna.045849.114 (PMC4174441; doi:10.1261/rna.045849.114)
Supplement: Supplemental Material [file supp_20_10_1593__index.html]

Drosophila Syncrip modulates the expression of mRNAs encoding key synaptic proteins required for morphology at the neuromuscular junction — Drosophila Syncrip modulates the expression of mRNAs encoding key synaptic proteins required for morphology at the neuromuscular junction — Supplemental Material 

# *Drosophila* Syncrip modulates the expression of mRNAs encoding key synaptic proteins required for morphology at the neuromuscular junction

## Supplemental Material

**Files in this Data Supplement:**

- Supp Figures.pdf
- Supp Table 1.xlsx
- Supp Material.doc
- Supp Table 3.xlsx
- Supp Table 2.xlsx
