# Supplementary figures and images for: Drosophila Syncrip modulates the expression of mRNAs encoding key synaptic proteins required for morphology at the neuromuscular junction
Source: RNA. 2014 Oct;20(10):1593–606. doi: 10.1261/rna.045849.114 (PMC4174441; doi:10.1261/rna.045849.114)

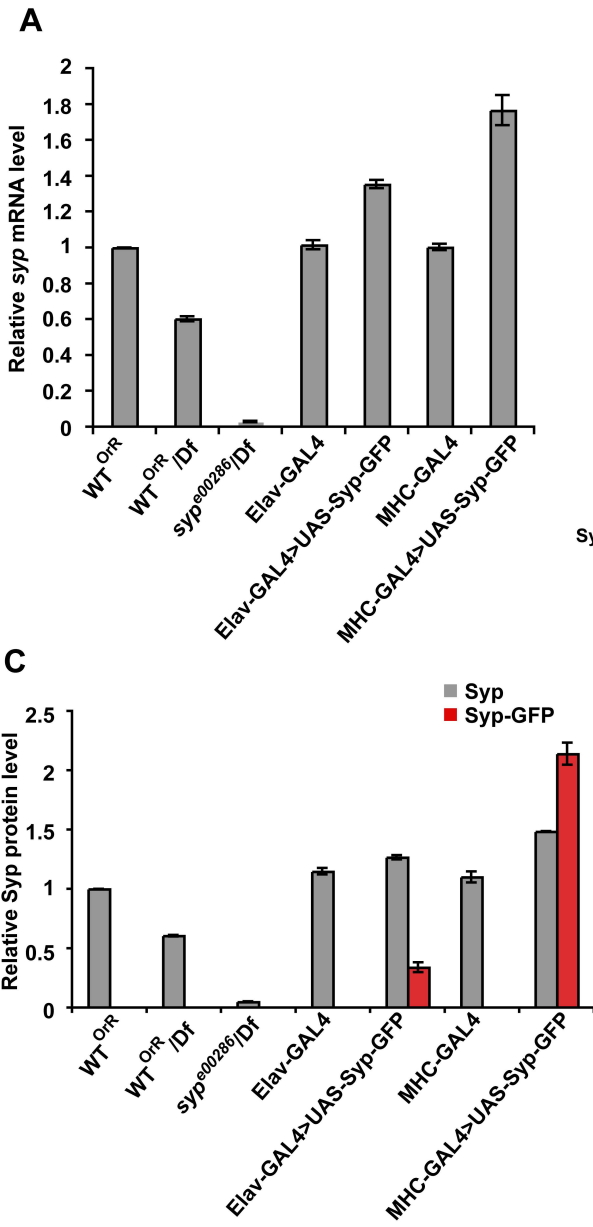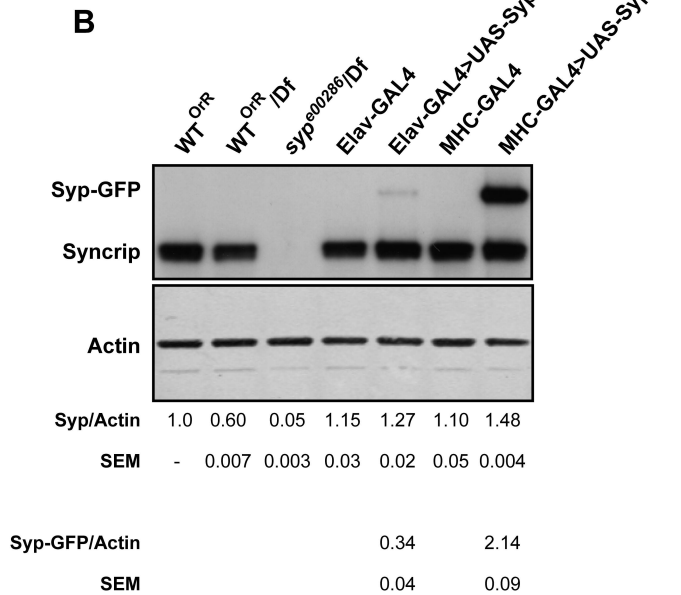

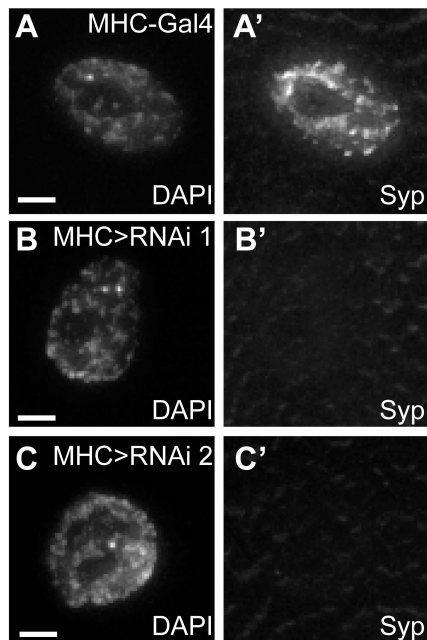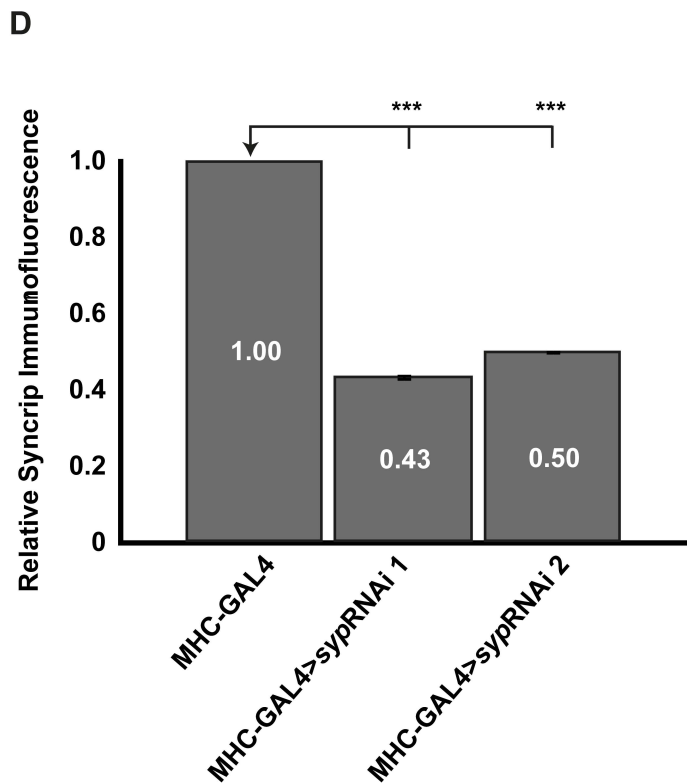

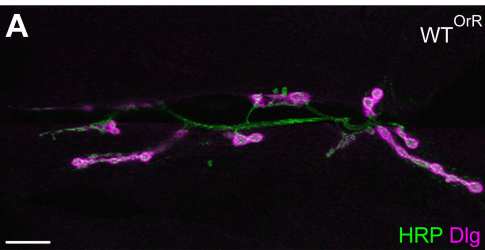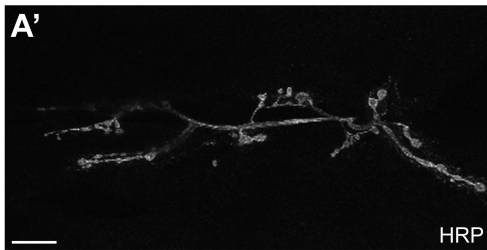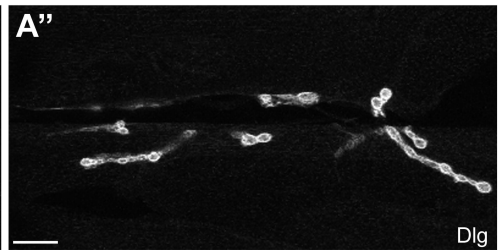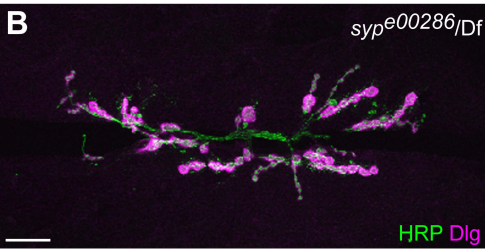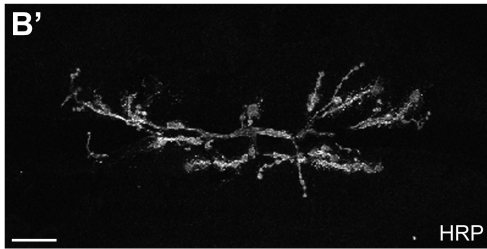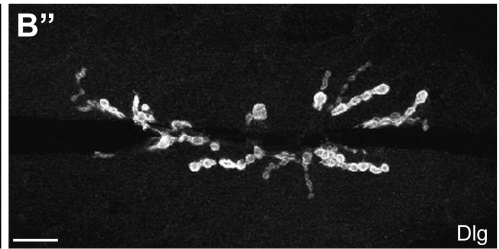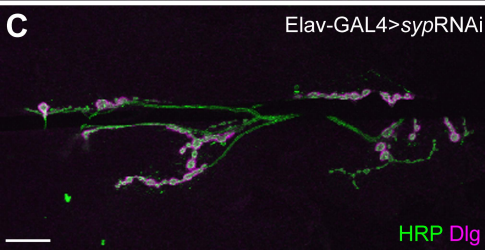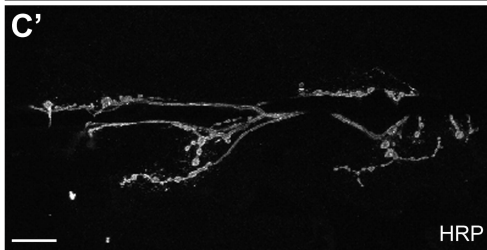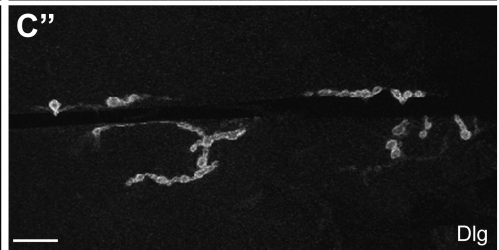

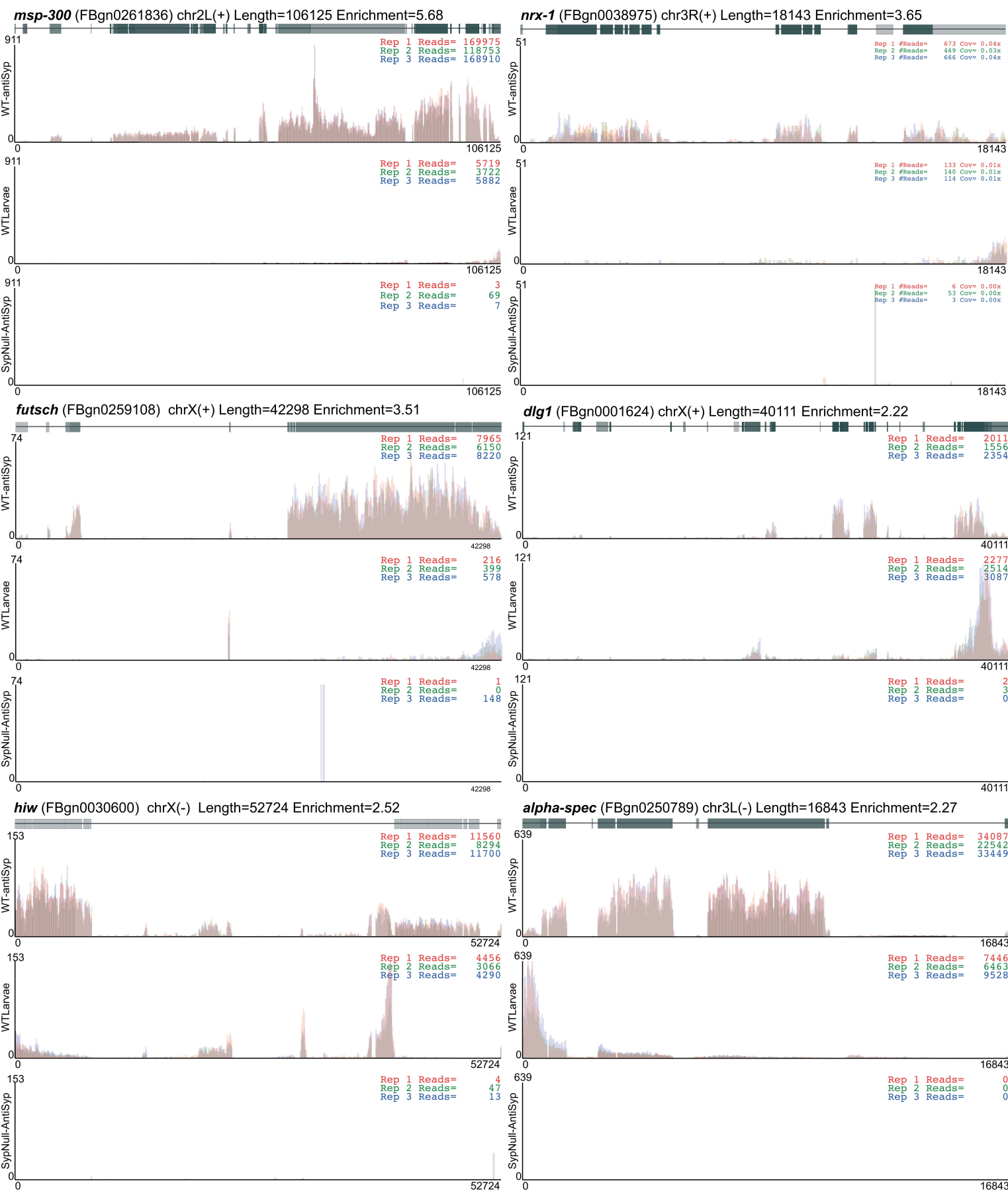

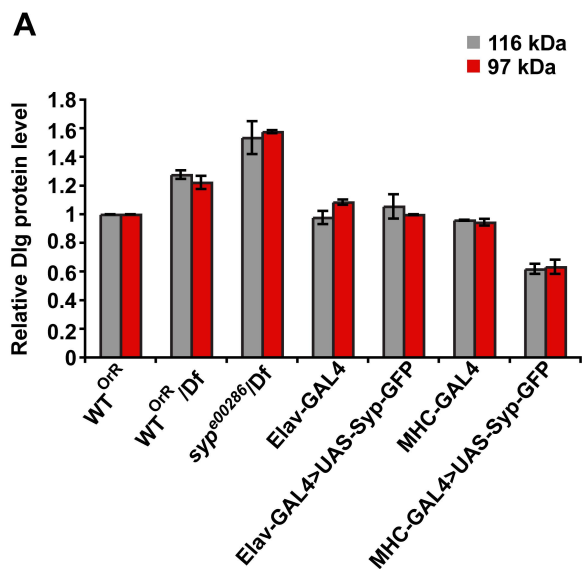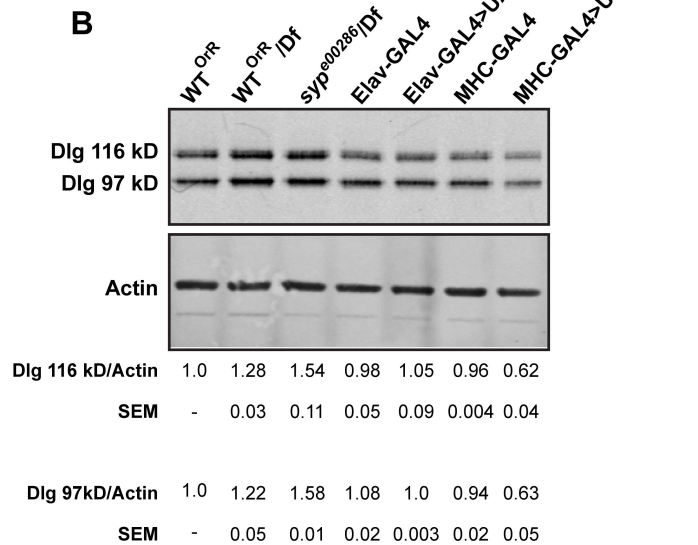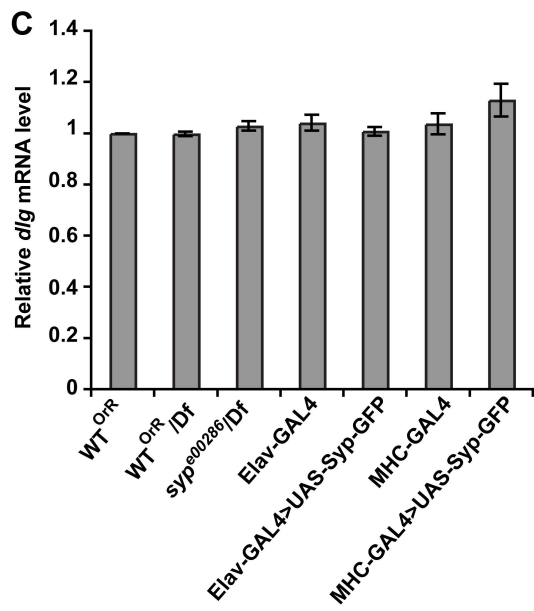

Supplementary Figure 6

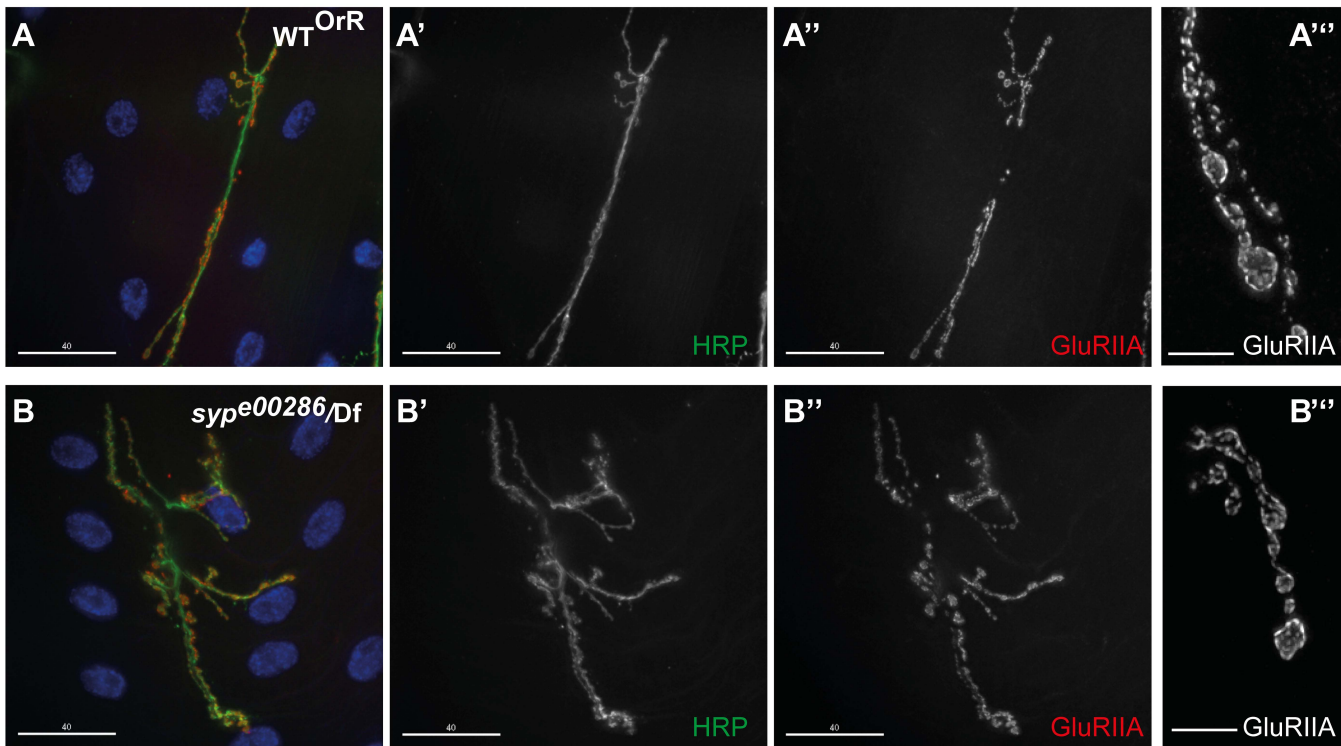

Supplement: Supplemental Material [file supp_045849.114_SuppFigs.pdf]
