# Supplementary material for: Drosophila Syncrip modulates the expression of mRNAs encoding key synaptic proteins required for morphology at the neuromuscular junction
Source: RNA. 2014 Oct;20(10):1593–606. doi: 10.1261/rna.045849.114 (PMC4174441; doi:10.1261/rna.045849.114)
Supplement: Supplemental Material [file supp_045849.114_REV_SupplementalMaterial.doc]

**Supplemental Information**

**Supplementary Table 1**

See Supplementary Table 1.xlsx

Ranked table of all transcripts identified by RNA-Seq to have a substantial number of reads where there was an enrichment due to anti-Syp immunoprecipitation from wild-type larval lysate, and not *syp* null larval lysate. Only transcripts above a defined cut off (>1; see Methods) for RNA enrichment are shown. The enrichment is calculated by comparing the log_2_ FPKM for WT-antiSyp to total WT larvae RNA-Seq.

**Supplementary Table 2**

See Supplementary Table 2.xlsx

**Functional Annotation Clustering of the Syp RIP-Seq dataset**

The top ten significant clusters from a DAVID GO Analysis for RNAs with a log_2_ enrichment > 1 (see Methods).

**Supplementary Table 3**

See Supplementary Table 3.xlsx

The coverage statistics for WT-antiSyp, total WT larvae and the *syp* null-antiSyp control RNA-Seq in triplicate (see Methods).

**Supplementary Figure 1. Levels of *syp* mRNA and Syp protein in wild-type, null mutant and Syp overexpressing larvae.**

(A) The levels of *syp* mRNA in third instar larval body wall muscle extracts of the indicated genotypes were quantified by RT-qPCR and normalized to rp49 mRNA. Error bars indicate the mean ± SEM from three independent experiments.

(B) Western blot showing relative levels of Syp and Syp-GFP in wild type, mutant and overexpression of Syp-GFP. *syp^e00286^*/Df shows no detectable protein.

(C) Quantitation of the Western blots, carried out with the Odyssey imaging system, using actin for normalization (see Materials and Methods for details). Data are shown as means ± SEM from three independent experiments.

**Supplementary Figure 2. Two RNAi insertions knock down *syp* in third instar larval muscle.**

(A-D) Expression of two independent RNAi insertions in muscle using the MHC-Gal4 driver (MHC-Gal4>*syp*RNAi 1 and 2) leads to ~50% knockdown of Syp in third instar larval muscles 6/7 of A3 hemisegments when compared to larvae containing only the MHC-Gal4 driver.

(A-C) Images are maximum intensity 5 μm projections, whilst (D) quantitative analysis was performed on the full z-stack. Error bars indicate the mean ± SEM. Statistical significance was calculated using Student’s t test (*, p<0.05; **, p<0.005; ***, p<0.001). Numbers of scored images was n=30 across 10 larvae. Scale bar 5 μm.

**Supplementary Figure 3. Pan-neuronal *syp* RNAi results in NMJ overgrowth.**

(A-C) Muscle 6/7 NMJs of A3 hemisegments labeled with anti-Dlg (magenta) and anti-HRP (green) antibodies in (A) wild-type (OrR), (B) mutant (*syp^e00286^*/Df), (C) pan-neuronal *syp* RNAi (Elav-GAL4>*syp*RNAi 2. Scale bar 20 µm.

**Supplementary Figure 4. RIP-Seq read coverage plots for selected transcripts.**

RNA-Seq read coverage plots aligned against gene models for *msp-300*, *futsch*, *hiw*, *nrx-1*, *dlg1* and *alpha-spec* for anti-Syp RIP from wild-type larvae (WT-antiSyp), total WT larvae and *syp* null larvae (SypNull-antiSyp). Reads are plotted for each of three RIP replicates in red, green and blue. The colour density of the exons represents the relative occurrence in the known FlyBase transcripts.

**Supplementary Figure 5. Syp regulates the level of DLG protein.**

(A-B) DLG protein levels in third instar larval body wall muscle extracts of the indicated genotypes were assessed by western blot analysis and quantified with the Odyssey imaging system, using actin for normalization. Data are shown as means ± SEM from three independent experiments. Note the two isoforms of DLG at 97 kDa and 116 kDa, probably representing DLGA and DLGS97 (Mendoza-Topaz et al., 2008). Levels of both isoforms are raised in the syp mutant and decreased upon overexpression of Syp in the muscle.

(C) The levels of *dlg1* mRNA in third instar larval body wall muscle extracts of the indicated genotypes were quantified by RT-qPCR and normalized to rp49 mRNA, showing no change in *dlg1* mRNA levels in syp mutants or Syp overexpression. Error bars indicate the mean ± SEM from three independent experiments. The total *dlg1* mRNA level is unaltered in all analyzed genotypes.

**Supplementary Figure 6. Loss of Syp does not affect GluRIIA expression.**

(A-B) Muscle 6/7 NMJs of A3 hemisegments labeled with anti-GluRIIA (red) and anti-HRP (green) antibodies in (A) wild-type (OrR), and (B) mutant (*syp^e00286^*/Df). Scale bar A-A’’ and B-B’’ 40 µm. Scale bar A’’’ and B’’’ 5 µm.

**Supplemental Materials and Methods**

**Antibody stocks and markers**

| Name | Raised in | Application | Dilution | Supplier |
| --- | --- | --- | --- | --- |
| anti-Syp | Guinea Pig | WB  IF  IP | 1/20000  1 μg/μl packed Protein A beads | Eurogentec |
| Control pre-immune serum | Guinea Pig | IP | 1 μg/μl packed Protein A beads | Eurogentec (for use with corresponding anti-Syp |
| anti-MSP-300 | Guinea Pig | WB  IF | 1/100 | Gift from Talila Volk |
| anti-Syd-1 | Rabbit | WB | 1/250 | Gift from Stephan Sigrist |
| anti-Hiw | Mouse | WB | 1/100 | DSHB |
| anti-Nrx | Goat | WB | 1/20 | Sigma |
| anti-actin | Rabbit | WB | 1/50 | Sigma |
| anti-tubulin | Mouse | WB | 1/100 | Sigma |
| anti-Discs large 4F3, concentrate | Mouse | WB  IF | 1/5000  1/500 | DSHB |
| anti-GluRIIA 8B4D2, concentrate | Mouse | IF | 1/200 | DSHB |
| anti-mouse/rabbit/guinea-pig IgG ECL antibody, HRP-conjugated | Sheep | WB | 1/5000 | GE Healthcare Life Sciences |
| IRDye 800 anti-mouse IgG | Goat | WB | 1/5000 | Licor |
| IRDye 680 anti-rabbit IgG | Goat | WB | 1/5000 | Licor |
| IRDye 800 anti-rabbit IgG | Goat | WB | 1/5000 | Licor |
| IRDye 680 anti-mouse IgG | Goat | WB | 1/5000 | Licor |
| IRDye 680 anti-guinea-pig IgG | Donkey | WB | 1/5000 | Licor |
| IRDye 680 anti-goat IgG | Donkey | WB | 1/5000 | Licor |
| Alexa Fluor goat anti-mouse/rabbit/guinea-pig IgG | Goat | IF | 1/500 | Life Technologies |

**List of primers used for qRT-PCR analysis**

| Name | Primer sequence 5’-3’ |
| --- | --- |
| CG17838 F | GATCGTGATAGCGCCGTCGAAG |
| CG17838 R | CTCACGAGCACGCAGAATCTC |
| rp49 F | GCTAAGCTGTCGCACAAATG |
| rp49 R | TCCGGTGGGCAGCATGTG |
| dlg1 F | ATCCGCATAATAATGTAAACTACGACAGAA |
| dlg1R | ACTCATTATATAGGTTTAAATCAACG |
| lsp2 F | GTATGTGGTGGGTCTGGTGC |
| lsp2 R | CAGGACGACTCCCTGCATATCATC |
| lk6 F | GTACAAGCTGACTGGCGAGATTC |
| lk6 R | TACCAGGTAGAACTTCTCGTCATCC |
| ubi-p63E F | ATACATACATGCACACACTCGCAAC |
| ubi-p63E R | CGACGCCAACTTTGTTGAGAAAC |
| msp-300 F | TGCGCGATAAGGAGCAACAG |
| msp-300 R | ATGAGGAGCTGTTCCGTTTGG |
| futsch F | GACGACGAGGACCTGCTACTAG |
| futsch R | AGAAGAATTGATCGTGCCGTTG |
| hiw F | CACCAGCCTCATCTGCGATTAAG |
| hiw R | GAGTCCGGTGGCACCAAATC |
| nrx-1 F | GTACATGTACGATGGAGCGCTC |
| nrx-1 R | TGCATCGATACTTTGTCAGACAACC |
| alpha-spec F | GGTTCCCTGGAACAGCAACTG |
| alpha-spec R | CTGGTCAAGCTGATCCCACTG |
