# Supplementary material for: Drosophila Syncrip modulates the expression of mRNAs encoding key synaptic proteins required for morphology at the neuromuscular junction
Source: RNA. 2014 Oct;20(10):1593–606. doi: 10.1261/rna.045849.114 (PMC4174441; doi:10.1261/rna.045849.114)
Supplement: Companion Paper [file supp_20_10_1593_v2_index.html]

Drosophila Syncrip modulates the expression of mRNAs encoding key synaptic proteins required for morphology at the neuromuscular junction — Companion Paper 

# *Drosophila* Syncrip modulates the expression of mRNAs encoding key synaptic proteins required for morphology at the neuromuscular junction

## Companion Paper

Click to access companion paper  
(Halstead JM, Lin YQ, Durraine L, Hamilton RS, Ball G, Neely GG, Bellen HJ, Davis I. 2014. Syncrip/hnRNP Q influences synaptic transmission and regulates BMP signaling at the *Drosophila* neuromuscular synapse. *BiO* doi: 10.1242/bio.20149027)
